# *miR‐363‐3p* induces EMT via the Wnt/β‐catenin pathway in glioma cells by targeting *CELF2*

**DOI:** 10.1111/jcmm.16970

**Published:** 2021-10-12

**Authors:** Bo Fan, Bolun Su, Guoqiang Song, Xin Liu, Zhongjie Yan, Shuai Wang, Fuguang Hu, Jiankai Yang

**Affiliations:** ^1^ Department of neurosurgery The Second Affiliated Hospital Hebei Medical University Hebei China; ^2^ Department of urology The Second Hospital of Baoding Hebei China

**Keywords:** *CELF2*, EMT, glioma, *miR‐363‐3p*, Wnt/β‐catenin pathway

## Abstract

In our previous study, we reported that *CELF2* has a tumour‐suppressive function in glioma. Here, we performed additional experiments to elucidate better its role in cancer. The expression profile of *CELF2* was analysed by the GEPIA database, and Kaplan–Meier curves were used to evaluate the overall survival rates. Four different online databases were used to predict miRNAs targeting *CELF2*, and the luciferase assay was performed to identify the binding site. The biological effects of *miR*‐*363*‐*3p* and *CELF2* were also investigated *in vitro* using MTT, Transwell, and flow cytometry assays. Western blotting, qPCR, and TOP/FOP flash dual‐luciferase assays were performed to investigate the impact of *miR*‐*363*‐*3p* and *CELF2* on epithelial‐to‐mesenchymal transition (EMT) and the Wnt/β‐catenin pathway. The effect of *miR*‐*363*‐*3p* was also tested *in vivo* using a xenograft mouse model. We observed an abnormal expression pattern of *CELF2* in glioma cells, and higher *CELF2* expression correlated with better prognosis. We identified *miR*‐*363*‐*3p* as an upstream regulator of *CELF2* and confirmed its direct binding to the 3′‐untranslated region of *CELF2*. Cell function experiments showed that *miR*‐*363*‐*3p* affected multiple aspects of glioma cells. Suppressing *miR*‐*363*‐*3p* expression inhibited glioma cell proliferation and invasion, as well as promoted cell death via attenuating EMT and blocking the Wnt/β‐catenin pathway. These effects could be abolished by the downregulation of *CELF2*. Treatment with ASO‐*miR*‐*363*‐*3p* decreased tumour size and weight in nude mice. In conclusion, *miR*‐*363*‐*3p* induced the EMT, which resulted in increased migration and invasion and reduced apoptosis in glioma cell lines, via the Wnt/β‐catenin pathway by targeting *CELF2*.

## 1 INTRODUCTION

Diffuse glioma is the most common aggressive primary brain tumour that accounts for 80% of malignant intracranial tumours.^1^ An increase in the glioma diagnosis rate has been seen in recent years, from 5.9 of 100,000 people in 1973 to 6.61 of 100,000 people in 2016, probably because of the improved diagnostic sensitivity.^2^ According to the 2016 WHO CNS tumour classification criteria, gliomas are graded from grade I to grade IV.^3^ Although remarkable advances have been made in the stratification of prognostication and diagnosis, little progress has been achieved in the therapy, especially in the treatment of high‐grade glioma. To date, most patients with advanced glioma receive standard care that includes surgical resection followed by temozolomide plus radiation, but the 5‐year overall survival remains poor.^4^ The pathogenesis of glioma is multifactorial, and it is considered that both genetic and environmental factors contribute to its emergence, as in the cases of other complex diseases.^5^ Identification of the underlying pathogenic mechanisms involved in the initiation and progression of glioma is critical for developing effective treatments.

*CELF2* is an RNA‐binding protein belonging to the CELF family of proteins, which binds to double‐ or single‐stranded RNA and regulates the expression of thousands of transcripts.^6^, ^7^ Comprehensive genomic analysis has shown that RNA‐binding proteins, including *CELF2*, are predominantly downregulated in tumours compared with their expression in normal tissues and play a crucial role in tumour development.^8^ Guo et al.^9^ showed that *CELF2* inhibits ovarian cancer progression by stabilizing FAM198B. Piqué et al.^10^ pointed out that *CELF2* was targeted by promoter hypermethylation‐associated transcriptional silencing in human cancer, and the presence of *CELF2* hypermethylation was associated with shorter overall survival in patients with breast cancer. *CELF2* was also shown to suppress non‐small cell lung cancer growth through the PI3‐K pathway.^11^

MicroRNAs (miRNAs) are a family of endogenous, single‐stranded, small non‐coding RNAs that act as post‐transcriptional regulators of gene expression.^12^ By binding to the 3′‐untranslated regions (3‐UTR) of target mRNAs, miRNAs repress translation or accelerate the degradation of mRNA and thus regulate biological processes, including cellular differentiation, proliferation, and apoptosis.^13^, ^14^, ^15^ Accumulating evidence has shown that dysregulation of miRNA levels plays an important role in various types of cancer. Several studies on the effects of *miR*‐*363*‐*3p* have been conducted in various cancers, and they reached divergent conclusions. Mohamed et al. reported that *miR*‐*363*‐*3p* increased chemoresistance of an ovarian cancer line to taxane.^16^ Dorbna et al.^17^ demonstrated that upregulated *miR*‐*363*‐*3p* prevented apoptosis and promoted growth of leukemic cells *in vitro*. In contrast, *miR*‐*363* was shown to be a tumour suppressor in lung cancer, osteosarcoma, and colorectal cancer.^18^, ^19^, ^20^ At present, only two studies examined the role of *miR*‐*363*‐*3p* with glioma cells. Bi et al.^21^ reported that HNF1A‐AS1 promotes glioma cell growth by acting as a *miR*‐*363*‐*3p* sponge. Xu et al.^22^ reported that *miR*‐*363*‐*3p* promotes cell proliferation, protects against apoptosis, and enhances invasion by directly targeting PDHB in glioma cells. The mechanism behind *miR*‐*363*‐*3p* modulating glioma cells needs further investigation.

Hence, we focussed on the relationship between *CELF2* and *miR*‐*363*‐*3p* in glioma cells. We identified *miR*‐*363*‐*3p* as an upstream regulator of *CELF2* expression, evaluated its biological functions *in vitro* and *in vivo*, as well as investigated the pathways involved in these processes. For the first time, we provided evidence that *miR*‐*363*‐*3p* induces the epithelial‐to‐mesenchymal transition (EMT) in glioma cells via the Wnt/β‐catenin pathway by targeting *CELF2*. Our findings suggest that *miR*‐*363*‐*3p* may be a novel therapeutic target for glioma therapy.

## 2 MATERIALS AND METHODS

### 2.1 Tissue specimens

Eighteen tumour tissue specimens, including 11 grade III and 7 grade IV samples, were obtained from patients with glioma from the Department of Neurosurgery of the Second Affiliated Hospital of the Hebei Medical University between April 2018 and January 2019. Detailed information about the collected samples is shown in Table 1. Patients or their family members were informed of the tissue collection, and they all signed written consent forms before the surgery. The experimental procedures were reviewed by the Ethics Committee of the Second Affiliated Hospital of the Hebei Medical University. Specimens were collected and stored in liquid nitrogen immediately after tumour resection to avoid RNA degradation.

**TABLE 1:** Characteristics of tissue samples for qPCR

Gender	Age	Pathology	WHO grade^a^
Male	56	Anaplastic astrocytoma	III
Male	64	Anaplastic oligodendroglioma	III
Female	55	Anaplastic ependymoma	III
Male	61	Anaplastic astrocytoma	III
Male	67	Anaplastic astrocytoma	III
Female	53	Anaplastic ependymoma	III
Male	69	Anaplastic ependymoma	III
Female	51	Anaplastic oligodendroglioma	III
Female	62	Anaplastic oligodendroglioma	III
Female	58	Anaplastic astrocytoma	III
Male	44	Anaplastic oligodendroglioma	III
Male	55	Glioblastoma	IV
Male	64	Glioblastoma	IV
Female	71	Glioblastoma	IV
Male	60	Glioblastoma	IV
Female	75	Glioblastoma	IV
Male	58	Glioblastoma	IV
Male	63	Glioblastoma	IV

^a^ According to the 2016 world health organization classification of tumors of the central nervous system.

### 2.2 Cell culture and transfection

U87 and U118MG cell lines were purchased from the American Type Culture Collection (Manassas, VA, USA). Cells were cultured in DMEM‐H medium at 37°C in the atmosphere of 95% air and 5% CO_2_ in an incubator and sub‐cultured after 2–3 days. One day before transfection, the cells were washed with phosphate‐buffered saline (PBS), trypsinized, and resuspended. Then, 2 mL of the cell suspension was added to the culture dish and cultured for 24 h. To prepare compound solution A, 20 pmol of miR mimic/antisense oligonucleotides (ASO) was diluted in 100 μl of Opti‐MEM (Gibco BRL, Gaithersburg, MD, USA). To prepare compound solution B, 20 μL of Lipofectamine 2000 (Invitrogen, USA) was diluted in 100 μl of Opti‐MEM. Solution B was mixed with solution A to compound transfection solution. Five microlitres of transfection solution was added into each well and incubated with cells for 4 h. Then, 3 mL of DMEM‐H was added to each well, and the cells were incubated for 24–48 h. The sequences are listed in Table S1.

### 2.3 Online database analyses

miRDB (http://www.miRdb.org/cgi‐bin/search.cgi/), miRWalk (http://mirwalk.umm.uni‐heidelberg.de), PICTAR (https://pictar.mdc‐berlin.de), and TargetScan (http://www.targetscan.org/vert_72/) databases were used to predict potential miRNAs that could target *CELF2*. TIMER2.0 (http://timer.cistrome.org) was used to investigate *CELF2* expression patterns and mutation rates in different tissues. GEPIA2 (http://gepia2.cancer‐pku.cn/#index) and ACLBI (https://www.aclbi.com/static/index.html#/) were used to detect *CELF2* expression levels in normal brain tissue and glioma tissue, as well as to compare the overall survival rates of groups of patients with high and low *CELF2* expression.

### 2.4 RNA isolation and qRT‐PCR

RNA was extracted from tissues and cell lines using TRIzol reagent, according to the manufacturer's instructions. Total RNA (5 μl), oligo(dT) primer (100 pmol/μl), RNasin, and M‐MLV were mixed and reacted to prepare the reverse transcription template. The primer sequences used are listed in Supplementary Table S1. PCR was performed using the IO5 Real‐Time PCR System (Applied Biosystems, USA). Then, the SYBR Premix Ex Taq miRNA qPCR assay kit (Takara, Japan) was used for quantitative determination. DNA or miRNA expression was normalized to that of *U6*. The fold change values were determined using the 2^−ΔΔCt^ method.

### 2.5 Western blot

Cell lines or tissues were lysed in the RIPA buffer (Saierbio, Tianjin, China). Total protein in each sample was collected and measured. Sodium dodecyl sulphate–polyacrylamide gel electrophoresis (SDS‐PAGE) was performed according to the manufacturer's instructions. Total protein was degenerated by boiling for 5 min with a loading buffer and cooled to room temperature. Protein samples were loaded into SDS‐PAGE wells for electrophoresis at 100 V. After transferring, the Hybond nitrocellulose membrane was blocked with the western blotting buffer for at least 60 min before incubation with the primary antibody. After the exposure to the primary antibody, the membrane was washed three times with the western blotting buffer and incubated with the secondary antibody. GAPDH expression was used as loading control. Proteins were detected using chemiluminescence substrates.

### 2.6 Luciferase assay

*CELF2* 3′‐UTR (positions 379–385: GUGCAAU) was cloned into the pmirGLO vector (Saierbio, Tianjin, China) to create a wild‐type (WT) pmirGLO‐CELF2‐3′‐UTR. The base sequence was mutated from GUGCAAU to CACCGAC to create a mutated (MUT) pmirGLO‐CELF2‐3′‐UTR. U87 cells were co‐transfected with the WT pmirGLO‐CELF2‐3′‐UTR or MUT pmirGLO‐CELF2‐3′‐UTR and *miR*‐*363*‐*3p* mimic or negative control (NC) using the Lipofectamine 2000 transfection system (Invitrogen, USA). The luciferase assays were performed using the Dual‐Luciferase Reporter Assay kit (Beyotime, China). An F200/M200 microplate fluorescence reader (Tecan Infinite) was used to detect luciferase activity under dark conditions. The relative luminescence unit value of Renilla luciferase was used as an internal reference.

### 2.7 MTT assay

Cells in the logarithmic growth phase were treated by trypsin and centrifuged to obtain a compound cell suspension of 1 × 10^4^/ml. Then, 100 μl of the suspension was added to four 96‐well plates and incubated for 0, 24, 48, and 72 h, respectively, under 5%CO_2_ at 37°C. Every 24 h, we took out one plate, added 20 μl of the MTT reagent (5 mg/ml) into each well, and incubated the plate for another 4 h. The supernatant was removed, and 150 μl of DMSO was added to each well to dissolve the precipitate. The absorbance value was measured at 570 nm using a microplate reader (BioTek, Winooski, VT, USA) within 10 min after the addition of DMSO. All experiments were repeated three times, and the data are presented as the mean ±standard deviation.

### 2.8 Transwell assay

BD BioCoat Matrigel (BD Biosciences, Bedford, MA, USA) was diluted at a ratio of 1:8 and used to coat the Transwell chambers (Corning, USA). The cells were starved for 12 h, trypsinized, and resuspended in serum‐free medium containing bovine serum albumin. Then, 100 μl of the cell suspension (1 × 10^5^ cells) was added to the Transwell chambers, and 600 μl of the medium containing 20% foetal bovine serum was added to the lower chambers. After a 24‐h incubation, the cells that passed through the Matrigel were fixed with 20% methanol. After crystal violet staining for 15 min, the cells were counted and photographed under a microscope (IX73, OLYMPUS, Japan) in three random fields.

### 2.9 Cell apoptosis assay

The cells were trypsinized and centrifuged at 1500 rpm for 3 min. After removing the supernatant, the cells were washed with PBS and resuspended. An aliquot containing 1 × 10^5^ cells was collected and mixed with 500 μl of the binding buffer. Then, 5 μl of annexin V and 5 μl of 7‐aminoactinomycin D (annexin V/7‐AAD Apoptosis Detection Kit, KeyGen, Nanjing, China) were added to the cell suspension. After a 15‐min incubation, the suspension was cooled on ice and analysed by flow cytometry.

### 2.10 Cell cycle assay

The cells were centrifuged and washed with precooled PBS before supernatant removal. After centrifugation, the cells were resuspended in 250 μl of cold PBS, added to 750 μl of ethanol, and fixed at −20°C overnight. The cells were centrifuged, washed with PBS, and added to 300 μl of PBS containing propidium iodide and RNase A and incubated for 30 min at 4°C. The samples were then investigated by flow cytometry.

### 2.11 *In vivo* experiments

Female BALB/c nude mice that were 5 weeks old and weighed approximately 18 g were purchased from Sepeifu (Beijing) Biotechnology Corp. Both the experimental and control groups contained six randomly assigned mice. The cells expressing ASO‐*miR*‐*363*‐*3p* or ASO‐NC were screened, cultured, and trypsinized. The cell density was adjusted to 1 × 10^7^/ml by PBS. The suspension was centrifuged and mixed with 80 μl of the serum‐free medium. To induce tumour growth, we injected 1 × 10^6^ transfected cells in 100 μl of DMEM‐H (Gibco BRL, Gaithersburg, MD, USA) into the backs of the mice. Mouse weight was measured before the injection. After the tumour was palpable (5 days after the injection), mouse weight was measured weekly. Tumour length (*L*) and width (*W*) were measured every 3 days. Tumour volume (*V*) was estimated using the following formula: *V* = 1/2*LW* ^2^. Endpoint was set at 4 weeks after inoculation. After the mice reached the experimental endpoint, they were euthanized by an intraperitoneal injection of 100 mg/kg sodium pentobarbital. The mice were checked for breathing and heartbeat status for 5 min to confirm death. Then, the tumours were dissected and weighed. Tumour tissues were sectioned for immunohistochemical staining. Experimental procedures were approved by the Animal Ethics Committee of The Second Affiliated Hospital of the Hebei Medical University (Approval Letter No.2021‐AE032).

### 2.12 Immunohistochemistry (IHC) staining

Before staining, the slides were soaked in the solution of potassium dichromate and concentrated H_2_SO_4_ to remove silica gel and smooth the slide surface. Then, the slide surface was coated with poly‐L‐lysine to increase tissue adherence. Tissue samples were embedded in liquid paraffin, sliced into 5‐μm‐thick sections, and then spread out on the slides. We used xylene for deparaffinization and H_2_O_2_ to remove endogenous peroxidases. Samples were sequentially incubated with primary and secondary antibodies and processed with the DAB Horseradish Peroxidase Colour Development Kit according to the manufacturer's instructions. The samples were stained with haematoxylin and observed under a microscope (IX73, OLYMPUS, Japan).

### 2.13 Statistical analysis

Statistical significance analysis was performed using the GraphPad Prism 8 software. *CELF2* expression levels in glioblastoma multiforme (GBM), lower grade glioma (LGG), and normal brain tissue from the TCGA were compared using the Kruskal–Wallis test. The survival curves were analysed using the Kaplan–Meier method. The correlation between the expression of *CELF2* and miRNAs in tissue samples was determined using the Spearman's rank correlation coefficient. The Student's *t* test was used to determine the significance of differences between the two groups in pairwise comparisons. All results are shown as the mean ±standard error of the mean. Effects were considered statistically significant if *p* < 0.05. All assays were repeated in triplicate.

## 3 RESULTS

### 3.1 Low expression of the *CELF2* gene in GBM samples

**FIGURE 1:**
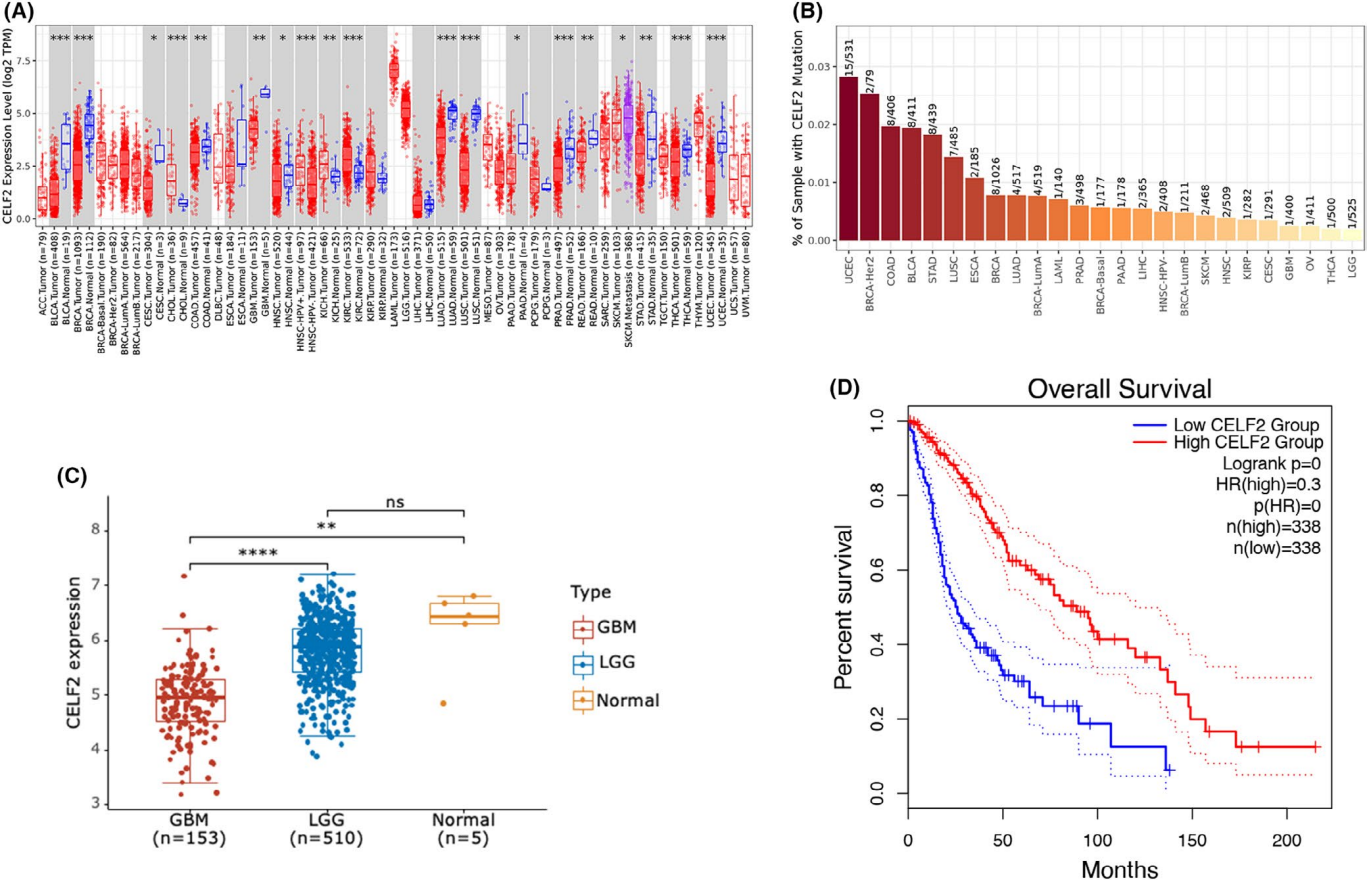
*CELF2* is low expsressed in glioma cells and higher *CELF2* expression suggests better prognosis. (A) *CELF2* had different expression levels in various types of cancer. (B) *CELF2* mutation rate was low in various types of cancer. (C) *CELF2* expression in GBM, LGG, and normal brain tissue. (D) Overall survival rate in glioma patients with low (*n* = 338) and high (*n* = 338) *CELF2* expression by Kaplan–Meier curves, *p* < 0.05. Data are expressed as mean ±SEM, **p < 0.001, ****p < 0.00001

We analysed *CELF2* expression levels and mutation rates in various cancer types. As shown in Figure 1A, 1B, *CELF2* was expressed at different levels in cancer and normal tissues but was not extensively mutated in cancer. Figure 1C shows lower *CELF2* expression level in GBM samples than in LGG and normal brain tissue samples. The mean expression level in LGG was lower than that in normal brain tissue, but the difference was not statistically significant. The Kaplan–Meier survival analysis showed that higher *CELF2* expression was associated with better prognosis (Figure 1D).

### 3.2 *miR*‐*363*‐*3p* directly suppresses *CELF2* expression by binding to its 3′‐UTR

**FIGURE 2:**
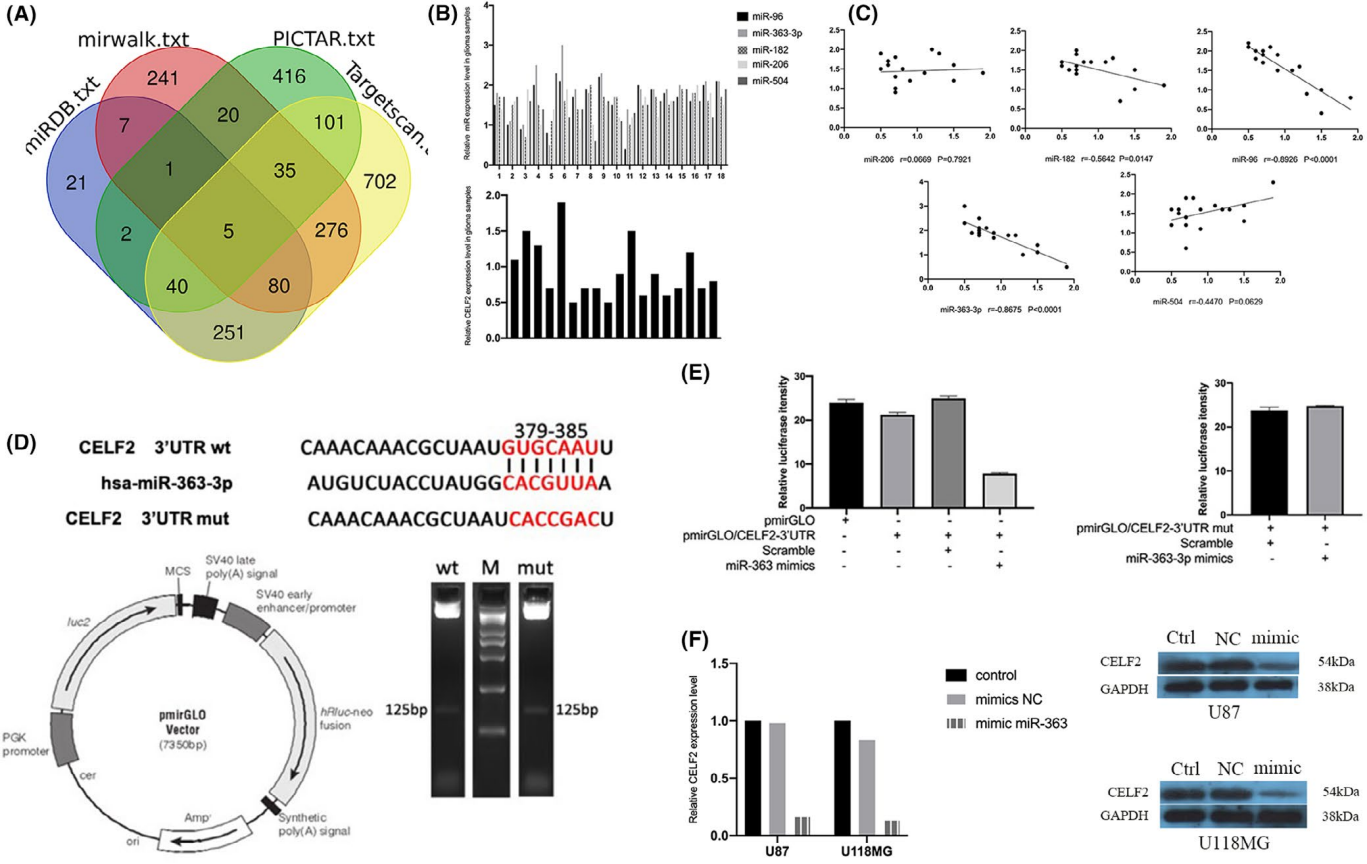
*miR*‐*363*‐*3p* directly binds to *CELF2* 3’‐UTR site. (A) A Venn diagram was utilized to search for miRNA which might target *CELF2*. (B) Expression levels of five candidate miRNAs and *CELF2* in 18 glioma samples were measured by qRT‐PCR. (C) The expression relations were determined by Spearman correlation test. (D) Predicted binding sites of *miR*‐*363*‐*3p* and *CELF2* 3’‐UTR were obtained from TargetScan. Vectors were constructed for *miR*‐*363*‐*3p* wt/mut. Enzyme‐digested products were agarose gel electrophoresed (AGE). (E) Luciferase activity was decreased only when *miR*‐*363*‐*3p* mimic and 3’‐UTR of *CELF2* were co‐transfected. (F) *CELF2* levels in U87 and U118MG cell lines were measured by western blot and qRT‐PCR after transfection of *miR*‐*363*‐*3p* mimic

To determine which miRNAs are targeting *CELF2*, we used online tools TargetScan, miRWalk, miRDB, and PicTar to predict candidates. We screened out five candidate miRNAs that were revealed in all four subsets (Figure 2A). We measured the expression of *CELF2* and five candidate miRNAs by qRT‐PCR in 18 glioma samples collected from the Department of Neurosurgery of the Second Affiliated Hospital of the Hebei Medical University (Figure 2B). We calculated the Spearman's rank correlation coefficient to analyse the relationship between expression levels of *CELF2* and each of the candidate miRNAs. We found that expression levels of *miR*‐*96* and *miR*‐*363*‐*3p* had a significant negative relationship with that of the *CELF2* mRNA. After referring to the literature, we selected *miR*‐*363*‐*3p* for further investigation (Figure 2C). We identified one conserved binding site for *miR*‐*363*‐*3p* in the 3′‐UTR of *CELF2* by TargetScan and constructed a vector for *miR*‐*363*‐*3p* wt/mut (Figure 2D). To determine whether a targeted combination existed, we conducted the dual‐luciferase assay. As shown in Figure 2E, increased expression of *miR*‐*363*‐*3p* inhibited luciferase activity. When the *CELF2* 3′‐UTR was mutated, the *miR*‐*363*‐*3p* mimic no longer affected luciferase activity (Figure 2E). Hence, we confirmed that *miR*‐*363*‐*3p* could directly bind to the 3′‐UTR of *CELF2* mRNA. Furthermore, when we increased the expression level of *miR*‐*363*‐*3p* in glioma cell lines, we observed decreased level of *CELF2* mRNA (Figure 2F). These results indicated that *miR*‐*363*‐*3p* suppressed post‐transcriptionally *CELF2* mRNA expression.

### 3.3 Effects of *miR*‐*363*‐*3p* on biological properties of glioma cell lines

**FIGURE 3:**
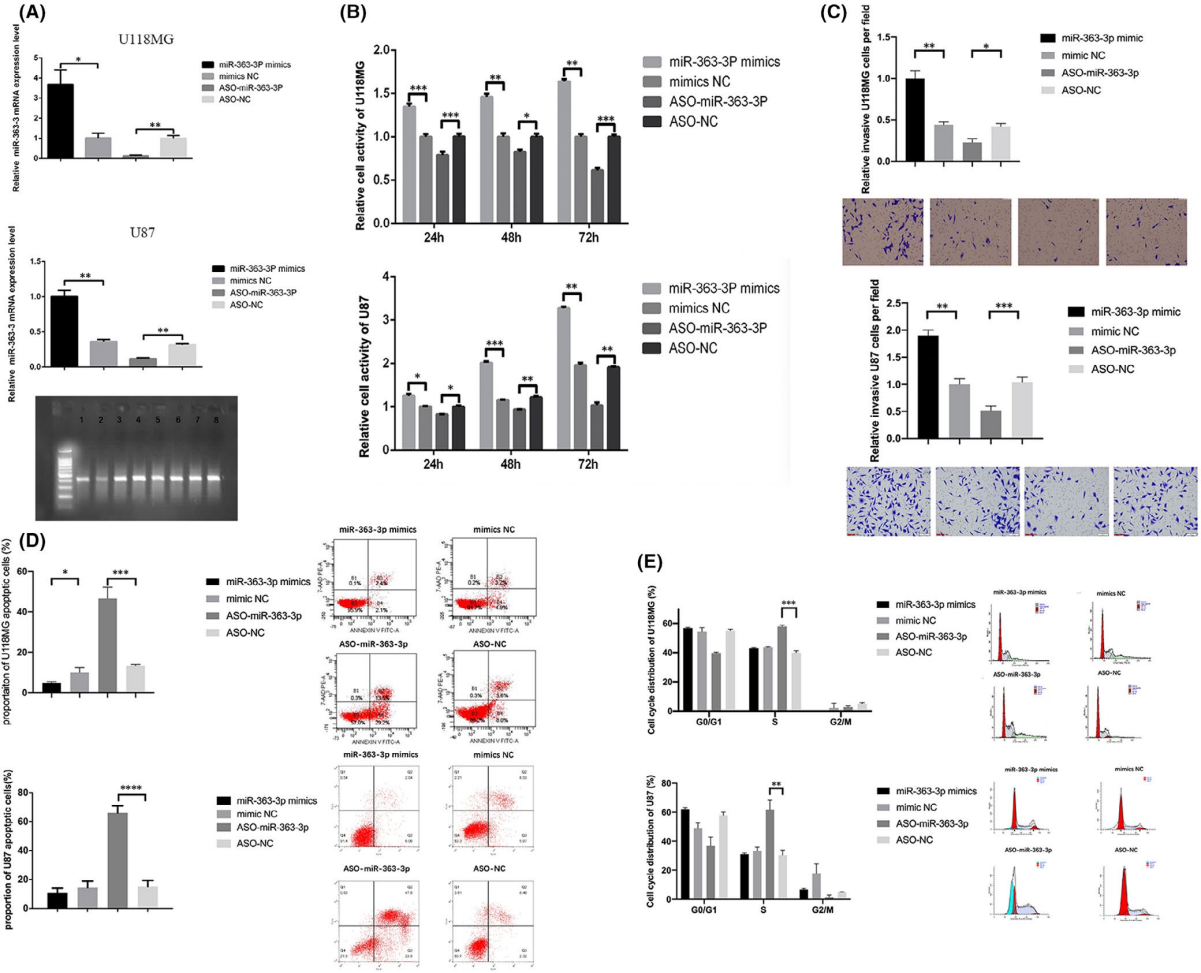
*miR*‐*363*‐*3p* affects biological behaviour of glioma cell lines by targeting *CELF2*. (A) qRT‐PCR test showed *miR*‐*363*‐*3p* mimic and ASO‐*miR*‐*363*‐*3p* were both effective in modulating *miR*‐*363*‐*3p* expression level. In AGE result, line 1–4 was for U118MG *miR*‐*363*‐*3p* mimic/mimics NC/ASO‐*miR*‐*363*‐*3p*/ASO‐NC, respectively, and line 5–8 was for U87 *miR*‐*363*‐*3p* mimic/mimics NC/ASO‐*miR*‐*363*‐*3p*/ASO‐NC, respectively. (B) Data were collected at 24, 48, and 72 h after incubation. MTT test showed that *miR*‐*363*‐*3p* mimic could increase cell proliferation, whilst *miR*‐*363*‐*3p* ASO could inhibit proliferation. (C) The photographs captured by microscope (×100) showed the cells stained by crystal violet after invading through the Matrigel membrane. Each column represented the mean number of cells in three randomized fields. (D) Cell apoptosis was tested by flow cytometry. The result showed *miR*‐*363*‐*3p* mimic impeded apoptosis and *miR*‐*363*‐*3p* ASO promoted cell death. (E) Cell cycle was tested by flow cytometry. Transfection of miR‐363‐3p ASO resulted in increased S phase, compared to control group. No significant difference was observed after *miR*‐*363*‐*3p* mimic transfection. Data are shown as means ±SEM. *p < 0.01, ***p < 0.0001, ****p < 0.00001, *n* = 3

To elucidate the role of *miR*‐*363*‐*3p* in glioma cells, we performed a series of *in vitro* experiments. Figure 3A shows that the expression of *miR*‐*363*‐*3p* could be efficiently modulated by transfecting glioma cells with mimic or ASO. We found that miR‐363‐3p mimic significantly increased the light absorbance by cell cultures treated with MTT in 24, 48, and 72 h after the transfection. In contrast, transfection with ASO‐*miR*‐*363*‐*3p* significantly decreased the light absorbance by glioma cell cultures, indicating lower amount of the formazan product and, therefore, lower number of viable cells (Figure 3B). In the Transwell assay, the *miR*‐*363*‐*3p* mimic group was more aggressive than the NC mimic group, whereas the ASO‐*miR*‐*363*‐*3p* group manifested a lower mobility and invasiveness than the ASO‐NC group (Figure 3C). Flow cytometry was performed to analyse the effect of *miR*‐*363*‐*3p* on cell apoptosis. Transfection of U87 and U118MG cells with *miR*‐*363*‐*3p* mimic reduced the number of apoptotic cells, whereas the transfection with ASO‐*miR*‐*363*‐*3p* increased their number (Figure 3D). Furthermore, we observed that treatment with ASO‐*miR*‐*363*‐*3p* significantly increased the number of cells in the S phase in both cell lines, indicating that repressing *miR*‐*363*‐*3p* led to S arrest of the cell cycle. When the cells were transfected with *miR*‐*363*‐*3p* mimic, no significant alterations in the G0/G1 and G2/M phases in either cell line were observed (Figure 3E). These data showed that *miR*‐*363*‐*3p* could affect cell apoptosis and that downregulation of *miR*‐*363*‐*3p* expression induced S arrest of cell growth.

### 3.4 Downregulation of *CELF2* expression eliminates the effects of ASO‐*miR*‐*363*‐*3p* on glioma cell lines

**FIGURE 4:**
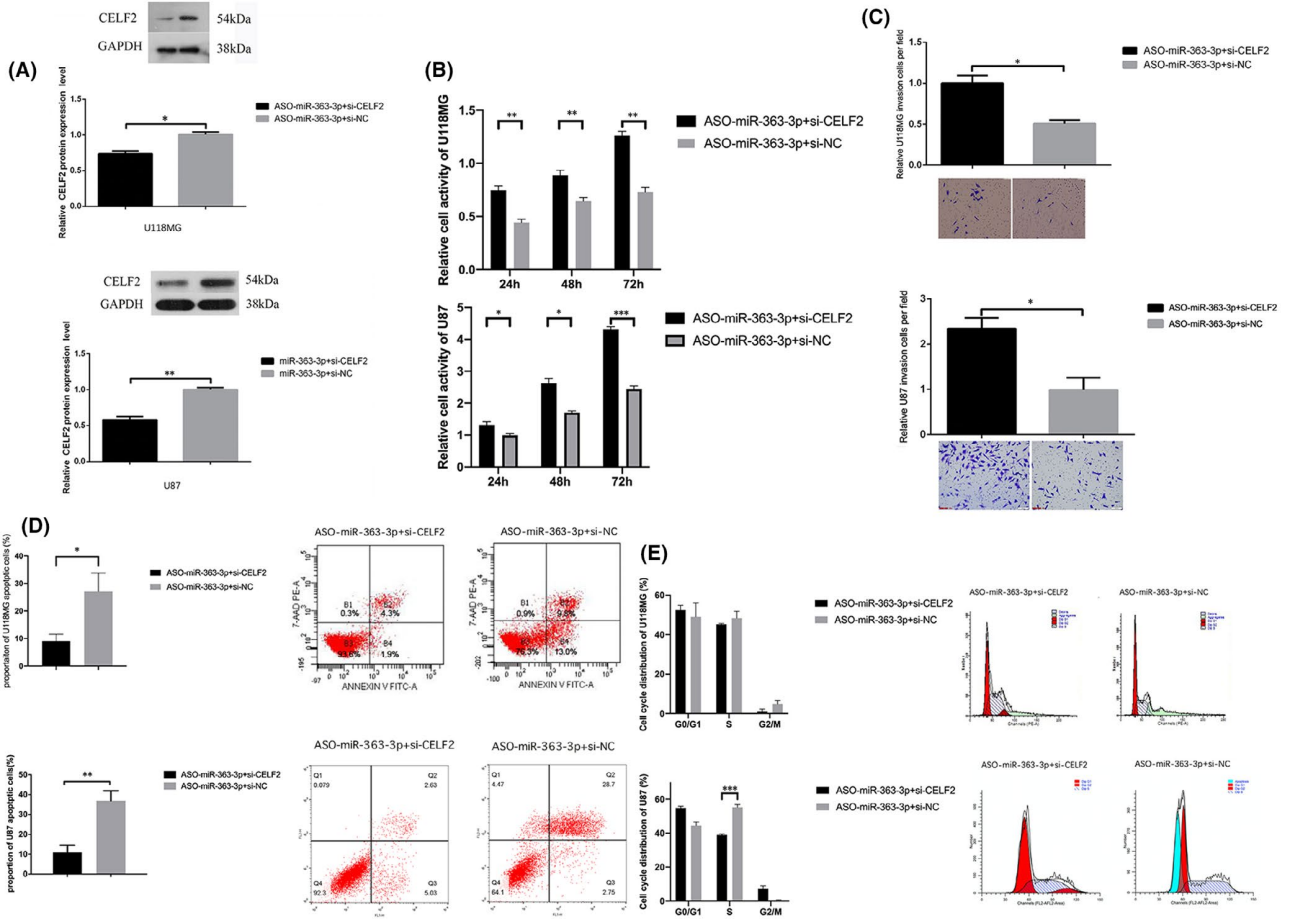
*CELF2* siRNA rescued ASO‐miR‐363‐3p‐induced cellular phenotypes in glioma cells. (A) *CELF2* level was measured after transfection of *CELF2* siRNA. (B) Cell activity was measured after co‐transfection with si‐*CELF2* and ASO‐miR‐363‐3p. (C) Cell proliferation was examined after co‐transfection with *CELF2* siRNA and AS0‐miR‐363‐3p. (D) Apoptosis was detected in U118MG and U87 cells. (E) Cell cycle was determined in U118MG and U87 cells. Data are shown as means ±SEM. *p < 0.01, **p < 0.001, ***p < 0.0001, *n* = 3

From the above experiments, we concluded that *miR*‐*363*‐*3p* is critical for glioma development. We subsequently designed rescue assays to confirm that *miR*‐*363*‐*3p* regulated glioma cell biological properties by targeting *CELF2*. *CELF2* siRNAs were transfected into glioma cells to specifically inhibit the increase in *CELF2* expression induced by ASO‐*miR*‐*363*‐*3p*. Figure 4A shows that *CELF2* siRNA effectively suppressed *CELF2* expression. Furthermore, treatment with *CELF2* siRNA completely abolished the effects of ASO‐*miR*‐*363*‐*3p* on U87/U118MG cells (Figure 4B–4D). In particular, treatment with *CELF2* siRNA significantly restored the S arrest induced by ASO‐*miR*‐*363*‐*3p* in U87 cells. However, this phenomenon was not observed in U118MG cells (Figure 4E). These results showed that *miR*‐*363*‐*3p* influenced the proliferation, invasion, and apoptosis of glioma cells by targeting *CELF2*.

### 3.5 *miR*‐*363*‐*3p* induces EMT and activates the Wnt/β‐catenin pathway by targeting *CELF2*

**FIGURE 5:**
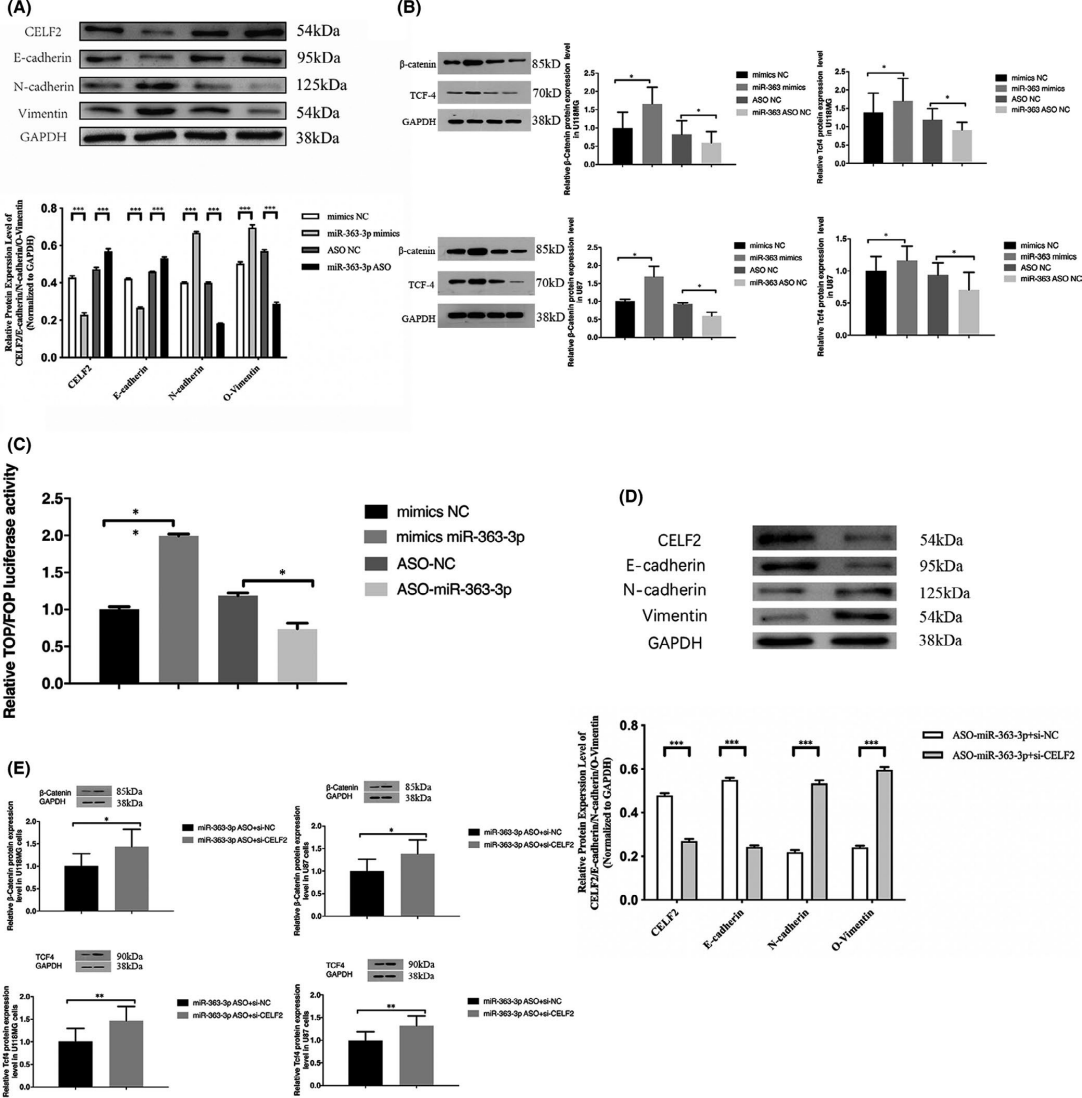
*miR*‐*363*‐*3p* induces EMT by targeting *CELF2* via Wnt/β‐catenin pathway. (A) Measurement of *CELF2*, E‐cad, N‐cad, and VIM expression in glioma cells after transfected with mimic or ASO. (B) Measurement of β‐catenin and TCF4 expression in glioma cells after transfected with mimic or ASO. (C) Activity of the Wnt/β‐catenin signalling pathway in glioma cell lines was recorded as TOP/FOP. (D) *CELF2*, E‐cad, N‐cad, and VIM expression in glioma cells were measured by qPCR ad Western blot. (E) qPCR and Western blot analyses of β‐catenin and TCF4 expression in glioma cells. Data are expressed as means ±SEM. *p < 0.01, **p < 0.001, ***p < 0.0001, *n* = 3

It is well established that the phenomenon EMT is strongly linked with tumorigenesis. We explored whether *miR*‐*363*‐*3p* affected the EMT. The expression levels of *CELF2* and EMT markers were examined by western blotting and qPCR. We observed that E‐cadherin (CDH1) was expressed at low levels, whereas expression levels of N‐cadherin and vimentin were upregulated after transfection of U87 cells with *miR*‐*363*‐*3p* mimic. Opposite results were observed after transfection with ASO‐*miR*‐*363*‐*3p* (Figure 5A). Further, we measured β‐catenin and TCF4 expression to assess the levels of the Wnt/β‐catenin signalling pathway activation. We found that expression levels of β‐catenin and TCF4 were increased in cells treated with *miR*‐*363*‐*3p* mimic and decreased in cells exposed to ASO‐*miR*‐*363*‐*3p* (Figure 5B). We performed the TOP flash/FOP flash dual‐luciferase reporter assay to further test whether the Wnt/β‐catenin signalling pathway was activated. We observed that cells treated with the *miR*‐*363*‐*3p* mimic exhibited significantly higher TOP/FOP luciferase activity than the control group. In contrast, cells transfected with ASO‐*miR*‐*363*‐*3p* had significantly lower activity than cells transfected with ASO‐NC (Figure 5C). In addition, treatment with si‐*CELF2* abrogated the effect of ASO‐*miR*‐*363*‐*3p* by reversing changes in expression levels of E‐cadherin, N‐cadherin, and vimentin, as well as in the expression of β‐catenin and TCF4 in both cell lines (Figure 5D, 5E). The result suggested that *miR*‐*363*‐*3p* induces EMT and activates the Wnt/β‐catenin pathway by targeting *CELF2*.

### 3.6 ASO‐*miR*‐*363*‐*3p* inhibits glioma cell growth *in vivo*

**FIGURE 6:**
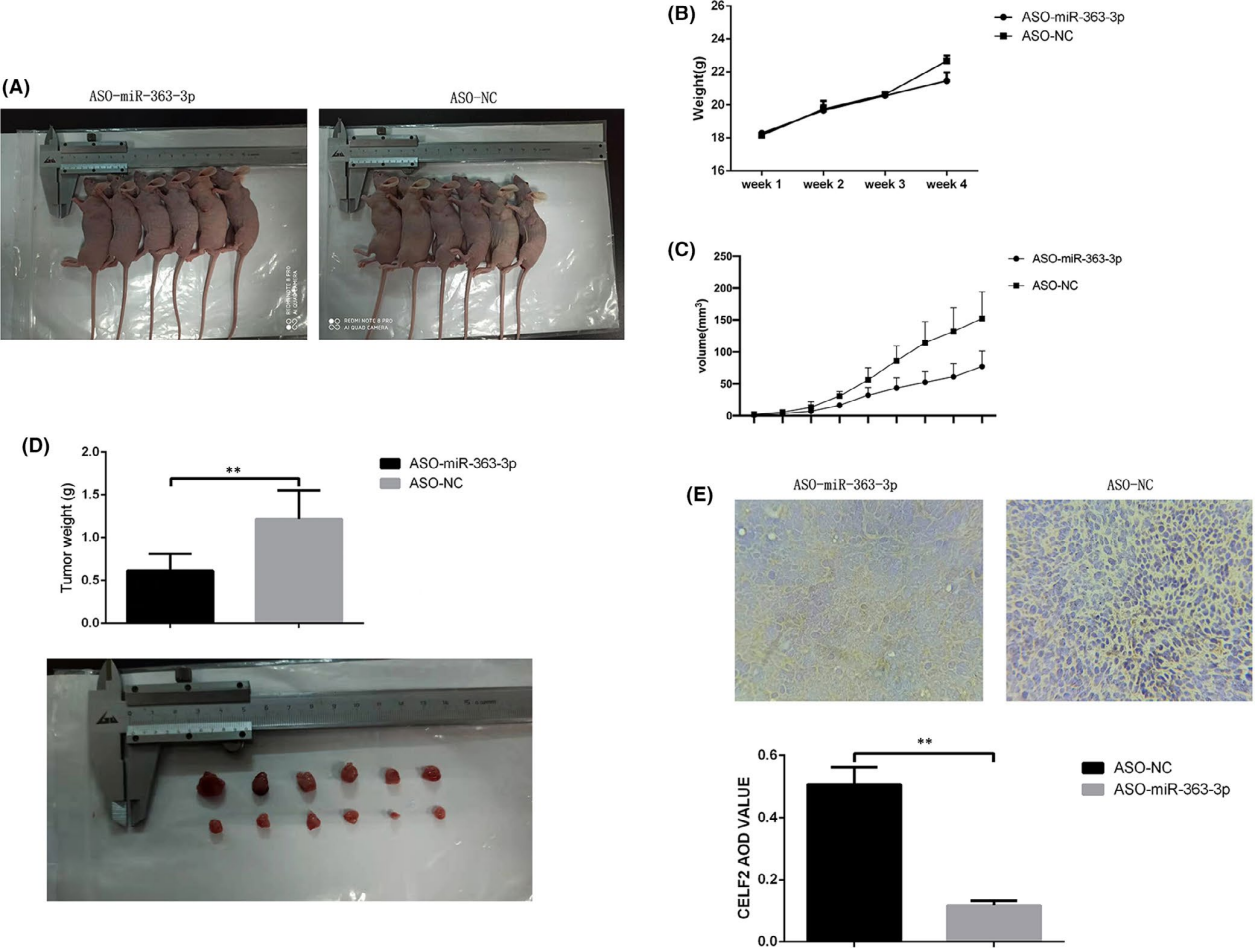
*miR*‐*363*‐*3p* promoted glioma cell proliferation and invasion ability in vivo. (A) Nude mice were injected on the back. (B) Body weight curve of nude mice after injection. (C) Tumour volume curve of nude mice after injection. (D) Tumours were peeled off after nude mice were euthanized. Tumour weight in experimental and control group was measured. (E) IHC staining for *CELF2* 4 weeks after implantation. Statistical analysis showed that *CELF2* expression level was significantly higher in ASO‐*miR*‐*363*‐*3p* group than in control group. Data are expressed as means ±SEM. *p < 0.05, **p < 0.01, ***p < 0.001, *n* = 3

To verify whether *miR*‐*363*‐*3p* affects glioma cells *in vivo*, we screened U87 cells transfected with ASO‐*miR*‐*363*‐*3p* (experimental group) or ASO‐NC (control group) and injected them into the back of nude mice to construct U87 xenograft models (Figure 6A). The experimental and control groups each contained nude mice of similar size and weight. The growth rate of tumours in the experimental group was significantly lower than that in the control group (Figure 6B, 6C). Four weeks later, nude mice were euthanized, and the tumours were dissected out. The volume and weight of tumours in the experimental group were significantly lower than those of the tumours in the control group (Figure 6D). IHC staining demonstrated that xenograft tumours derived from the ASO‐*miR*‐*363*‐*3p* transfected cells had significantly higher *CELF2* expression than tumours in the control group (Figure 6E). Thus, our data showed that ASO‐*miR*‐*363*‐*3p* inhibits glioma cell growth *in vivo*.

## 4 DISCUSSION

Glioma is the most common primary brain tumour that accounts for approximately 30% of all types of tumours in the central nervous system.^23^ miRNAs have been shown to function as tumour suppressors or proto‐oncogenes during tumorigenesis in various human cancers.^24^, ^25^ In our previous study, we demonstrated a tumour‐suppressive function of *CELF2* in glioma cells.^26^ This study was designed to uncover the role of *miR*‐*363*‐*3p* as a negative modulator of *CELF2* expression in the development of glioma.

By using an online database, we found that *CELF2* was expressed at low levels in most cancers, whereas its mutation rate was low. We hypothesized that *CELF2* expression is suppressed post‐transcriptionally during tumorigenesis. According to the data in the TCGA database, GBM had lower *CELF2* expression levels than LGG or normal brain tissue. Furthermore, *CELF2* levels negatively correlated with survival time. To determine upstream regulators of *CELF2* expression, we used four prediction tools and identified five miRNA candidates that were present in all prediction results. We measured expression levels of *CELF2* and each miRNA candidate in 18 glioma samples to determine their possible correlation. Spearman's test showed that levels of three of the identified miRNAs negatively correlated with *CELF2* expression, and we finally selected *miR*‐*363*‐*3p* for further study. The luciferase assay verified that *miR*‐*363*‐*3p* could bind to the 3′‐UTR of *CELF2*. It has been reported that *miR*‐*363*‐*3p* is involved in multiple processes during tumour development.^27^, ^28^, ^29^ We performed a series of experiments to confirm biological functions of *miR*‐*363*‐*3p*. We found that *miR*‐*363*‐*3p* affected cell proliferation, invasive ability, and apoptosis in the two tested glioma cell lines, as well as influenced tumour size and weight *in vivo*. Based on our experimental data, we concluded that *miR*‐*363*‐*3p* is important for glioma migration and recurrence. The results of the cell cycle experiment were somewhat confusing. As shown in Figure 3B, the proliferation of U87 and U118MG cells was decreased after their transfection with ASO‐*miR*‐*363*‐*3p*. In the experiments illustrated in Figure 3E, we found that the proportion of cells in the S phase was significantly increased in the cell cycle after transfection with ASO‐*miR*‐*363*‐*3p*, which meant that more cells were stuck in the S phase and could not proceed to the next phase to complete mitosis. Given that, we concluded that cell mitosis was arrested in the S phase by ASO‐*miR*‐*363*‐*3p*. However, the results in U87 and U118MG cells were contradictory (Figure 4E). One explanation of this discrepancy could be that U87 and U118MG have different mechanisms to regulate cell cycle. In U87 cells, *CELF2* plays a key role in regulating the cell cycle. *CELF2* siRNA significantly suppressed *CELF2* expression and thereby profoundly affected the cell cycle. However, the cell cycle of U118MG cells might be controlled by another protein, which is also targeted by *miR*‐*363*‐*3p*. Thus, ASO‐*miR*‐*363*‐*3p* led to the arrest in the S‐phase, but treatment with si‐*CELF2* could not reverse this effect.

In our previous study, we showed that *miR*‐*95*‐*3p* promoted cell proliferation and migration but inhibited apoptosis by targeting *CELF2*,^26^ i.e. acted in a similar fashion to *miR*‐*363*‐*3p*. Nevertheless, we noticed an interesting phenomenon. ASO‐*miR*‐*95*‐*3p* did not significantly change the cell cycle, whereas ASO‐*miR*‐*363*‐*3p* induced S arrest by targeting *CELF2* in U87 cells. We speculate that besides *CELF2*, miR‐95‐3p might target another gene, which is also crucial to the cell cycle in U87 cells. After transfection with ASO‐*miR*‐*95*‐*3p*, the levels of both *CELF2* and that unknown protein were increased, and the combined effect was that the cell cycle manifested no significant change.

Accumulating evidence indicates that EMT is closely involved with the metastatic ability of cancer cells, as it confers the loss of cell‐to‐cell adhesion and gain of migratory properties.^30^ In fact, EMT participates in various normal physiological processes, including embryonic development, organ fibrosis, tissue regeneration, but also regulates tumour stemness, tumour initiation and malignant progression, cell migration and invasion, intravasation to the blood, metastasis, and resistance to therapy.^31^, ^32^, ^33^ When EMT is activated, the expression of E‐cadherin decreases. In addition, increased expression levels of N‐cadherin and vimentin are also critical markers of the EMT process during tumour progression.^34^

The Wnt/β‐catenin signalling pathway regulates the EMT in several cancers. When activated, Wnt ligands bind to the LRP 5/6 co‐receptor and the Frizzled receptor. The latter protein interacts with the Dishevelled protein to suppress the activity of GSK‐3β, which inhibits β‐catenin degradation. Accumulating β‐catenin is released into the cytoplasm and gets translocated into the nucleus, where it binds to TCF/LEF and replaces the repressor protein Groucho/TLE.^35^ This results in the activation of TCF/LEF transcription and expression of genes encoding such proteins as AXIN2, JUN, MYC, cyclin D1, WISP, and SP5 (specificity protein transcription factor 5). Activation of these genes induces EMT and promotes tumour metastasis.

Activation of the Wnt/β‐catenin pathway is closely related to glioma development. Xu et al. reported that *miR*‐*133b* suppresses glioma growth via the Wnt/β‐catenin pathway.^36^ The Wnt/β‐catenin pathway can also activate miRNAs that affect glioma cell progression.^37^ By qPCR and western blotting, we verified that overexpression of *miR*‐*363*‐*3p* induced EMT by downregulating E‐cadherin and upregulating N‐cadherin and vimentin expression. Furthermore, *miR*‐*363*‐*3p* promoted expression of β‐catenin and TCF4, which play critical roles in the Wnt/β‐catenin pathway. Data on the TOP/FOP luciferase activity also confirmed that the Wnt/β‐catenin pathway was activated by *miR*‐*363*‐*3p*. When we treated cells with si‐*CELF2*, we found that changes in expression levels of E‐cadherin, N‐cadherin, and vimentin, as well as in the expression of β‐catenin and TCF4 in both cell lines by the effect of ASO‐*miR*‐*363*‐*3p*, were all abrogated. Thus, we concluded that *miR*‐*363*‐*3p* induces EMT via the Wnt/β‐catenin pathway by targeting *CELF2*.

*CELF2*, as an RNA‐binding protein, usually exerts its function of regulating protein transcription by binding mRNAs. Yeung et al.^11^ reported that *CELF2* interacts with PREX2 and reduces the association of PREX2 with PTEN. Guo et al.^9^ showed that *CELF2* expression increased the stability of its target, FAM198B, by binding to the AU/U‐rich elements in *FAM198B* 3′‐UTR in ovarian cancer. Therefore, it is reasonable to believe that there might be a protein downstream of *CELF2*, participating in this regulation process. Direct evidence is needed to demonstrate how *CELF2* interacts with the EMT or Wnt/β‐catenin pathway.

In conclusion, our experiments in glioma cell lines showed that *miR*‐*363*‐*3p* induced the EMT, which resulted in increased migration and invasion and reduced apoptosis via the Wnt/β‐catenin pathway by targeting *CELF2*. This evidence suggests that *miR*‐*363*‐*3p* may be a novel therapeutic target for glioma.

## CONFLICT OF INTEREST

The authors confirm that there are no conflicts of interest.

## AUTHOR CONTRIBUTIONS

**Bo Fan:** Conceptualization (lead); Formal analysis (lead); Writing‐original draft (lead); Writing‐review & editing (equal). **Bolun Su:** Data curation (equal); Formal analysis (supporting); Software (lead). **Guoqiang Song:** Investigation (equal); Writing‐review & editing (equal). **Xin Liu:** Investigation (equal); Writing‐review & editing (equal). **Zhongjie Yan:** Investigation (equal); Writing‐review & editing (equal). **Shuai Wang:** Resources (equal); Writing‐review & editing (equal). **Fuguang Hu:** Resources (equal); Writing‐review & editing (equal). **Jiankai Yang:** Investigation (equal); Project administration (equal); Supervision (equal); Writing‐review & editing (equal).

## Supporting information

Table S1

## Data Availability

The data that support the findings of this study are available from the corresponding author upon reasonable request.
