# Supplementary material for: miR‐363‐3p induces EMT via the Wnt/β‐catenin pathway in glioma cells by targeting CELF2
Source: J Cell Mol Med. 2021 Oct 12;25(22):10418–29. doi: 10.1111/jcmm.16970 (PMC8581338; doi:10.1111/jcmm.16970)
Supplement: Supplementary file 1 — Table S1 [file JCMM-25-10418-s001.docx]

Supplementary table S1

|  | *SEQUENCE* |
| --- | --- |
| miR-363-3p mimics | AAUUGCACGGUAUCCAUCUGUA |
| Mimics NC | UUCUCCGAACGUGUCACGUTT |
| ASO-miR-363-3p | UACAGAUGGAUACCGUGCAAUU |
| ASO-NC | CAGUACUUUUGUGUAGUACAA |
| CELF2 siRNA | CAGGCAUGAAUGCUUUACATT |
| U6 primer F | TGGGGTTATACATTGTGAGAGGA |
| U6 primer R | GTGTGCTACGGAGTTCAGAGGTT |
| miR-363-3p primer F | AATTGCACGGTATCCA |
| miR-363-3p primer R | CATGATCAGCTGGGCCAAGA |
| CELF2 primer F | AAACTAGCGGCCGCTAGTCAAACAAACGCTAATGTGCAATTT |
| CELF2 primer R | CTAGAAATTGCACATTAGCGTTTGTTTGACTAGCGGCCGCTAGTTT |
